# Validation and Application of a New Reversed Phase HPLC Method for *In Vitro* Dissolution Studies of Rabeprazole Sodium in Delayed-Release Tablets

**DOI:** 10.1155/2013/976034

**Published:** 2013-08-22

**Authors:** Md. Saddam Nawaz

**Affiliations:** Quality Assurance Department, ACI Ltd., Narayanganj 1400, Bangladesh

## Abstract

The purpose of this study was to develop and validate a new reversed phase high performance liquid chromatographic (RP-HPLC) method to quantify *in vitro* dissolution assay of rabeprazole sodium in pharmaceutical tablet dosage form. Method development was performed on C 18, 100 × 4.6 mm ID, and 10 **μ**m particle size column, and injection volume was 20 **μ**L using a diode array detector (DAD) to monitor the detection at 280 nm. The mobile phase consisted of buffer: acetonitrile at a ratio of 60 : 40 (v/v), and the flow rate was maintained at 1.0 mL/min. The method was validated in terms of suitability, linearity, specificity, accuracy, precision, stability, and sensitivity. Linearity was observed over the range of concentration 0.05–12.0 **μ**g/mL, and the correlation coefficient was found excellent >0.999. The method was specific with respect to rabeprazole sodium, and the peak purity was found 99.99%. The method was precise and had relative standard deviations (RSD) less than 2%. Accuracy was found in the range of 99.9 to 101.9%. The method was robust in different variable conditions and reproducible. This proposed fast, reliable, cost-effective method can be used as quality control tool for the estimation of rabeprazole sodium in routine dissolution test analysis.

## 1. Introduction

**Figure 1:**
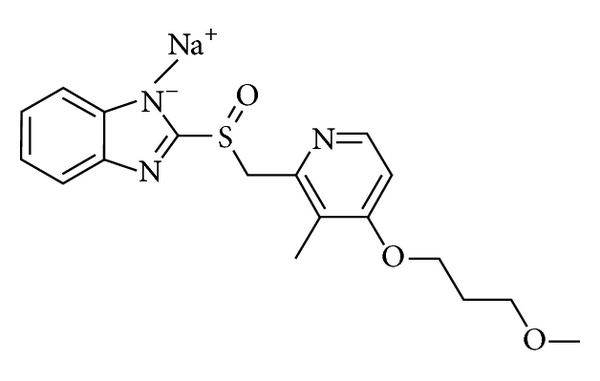
Chemical structure of rabeprazole sodium.

Since 1980s, proton pump inhibitors (PPIs) are the most potent inhibitors of gastric acid secretion and effective for treating all gastric acid-related disorders, including gastroesophageal reflux disease (GERD), peptic ulcer disease (PUD), and nonsteroidal anti-inflammatory drug- (NSAID-) induced gastropathy. Rabeprazole sodium (RPS) belongs to a class of PPIs that suppresses gastric acid secretion by specific inhibition of the enzyme system of hydrogen/potassium adenosine triphosphatase (H^+^/K^+^ ATPase) at the secretory surface of the gastric parietal cell. In contrast to the other PPIs, RPS is the most potent acid secretion inhibitor during first day of dosing [1]. RPS hinders gastric acid secretion up to the final steps [2]. It may also be used with an antibiotic to prevent gastric ulcer caused by infection *Helicobacter pylori (H. pylori)*. Rabeprazole sodium is chemically 2-[{(4)3-methoxypropoxy-3-methyl-2-pyridinyl} sulphinyl] 1-H benzimidazole sodium salt (Figure 1). It has an empirical formula of C_18_H_20_N_3_NaO_3_S, molecular weight of 381.43, and half-life of 1-2 hour. RPS is a white to yellowish crystalline solid and soluble in water, methanol, and acetonitrile but insoluble in ether and n-hexane. RPS is a substituted benzimidazole which stability is depending on pH. Like most other PPIs, it is rapidly degraded in acid medium to yield two main products, the sulfenamide and the benzimidazole sulphide, but it is more stable under basic condition.

Rabeprazole sodium is not available yet any of the pharmacopoeias. Methods that have been reported for the determination of RPS include HPLC [3–11], thin layer chromatography (TLC) [12], HPTLC [13, 14], UV spectrophotometry [15–17], LCMS [18], and voltametry [19]. And some methods have also been published for the quantitative analysis of RPS in combination formulations with other drugs [20, 21].

Although a number of articles had been published for the HPLC assay of RPS, they were mostly in combination with antiemetic drug domperidone or cholinergic drug itopride. There is no solution stability representing validated RP-HPLC method so far published to investigate dissolution assay of RPS. The aim of the present work was to develop a simple, precise, fast, and solution stability demonstrating RP-HPLC method for the determination of RPS in delayed-release (DR) tablet dosage form during *in vitro* dissolution studies. The authentication of the applicability of this developed method was validated according to the International Conference on Harmonization (ICH) Q2 (R1) and the United States Pharmacopeia (USP) [22, 23].

## 2. Experimental 

### 2.1. Reagents and Chemicals

The RPS standard and API were obtained from Metrochem API Private Limited, Hyderabad, India. AcipHex (Eisai Inc., NJ, USA) tablets (each DR tablet contains 20 mg rabeprazole sodium) were purchased commercially from the market. Acetonitrile HPLC grade (Scharlau, Spain), methanol HPLC grade (Scharlau, Spain), disodium hydrogen phosphate AR grade (Scharlau, Spain), phosphoric acid AR grade (Merck; Germany), hydrochloric acid (Scharlau, Spain) and Tris (hydroxymethyl) aminomethane AR grade (Scharlau, Spain) were used for analytical purposes. Ultrapure water was used to prepare the mobile phase, diluting solution and dissolution medium. Ultra pure water was prepared by using Labconco WaterPro PS (USA) purification system.

### 2.2. Instrumentation and Chromatographic Condition

Chromatographic separation was achieved by using Shimadzu (Japan) prominence LC-20AD high performance liquid chromatography, equipped with degasser PGU-20A 5, variable wavelength programmable diode array detector SPD-M20A, autosampler SIL-20 AC HT, and column oven CTO-10 A5 VP. The data was recorded using LC LabSolution software (Japan). ProntoSIL SC, C 18-ace-EPS, 100 × 4.6 mm ID, and 10 *μ*m particle size column (Bischoff, Germany), was used as the stationary phase. The column oven temperature was kept at ambient condition and the mobile phase flow rate was maintained at 1.0 mL/min. The detection was monitored at 280 nm. The injection volume was 20 *μ*L, and the run time was 4 min for each injection. Other instruments such as dissolution apparatus (Logan UDT-804-12, USA), pH meter (Jenway 3510, UK), electronic weighing balance (Mettler-Toledo, Switzerland) and ultrasonic bath (Clifton, UK) were also used. 

Dionex ultimate 3000 series (USA) HPLC and Chromeleon software (USA) were used during ruggedness study.

#### 2.2.1. Mobile Phase

To prepare buffer solution for mobile phase, 1.42 g of disodium hydrogen phosphate was dissolved in 800 mL of ultra pure water. Then the solution pH was adjusted to 7.6 ± 0.1 with orthophosphoric acid and volume up to 1000 mL with ultra pure water.

Finally a mixture of above prepared buffer and acetonitrile (ACN) at a ratio of 60 : 40 (v/v) was used as mobile phase. The prepared buffer and acetonitrile (ACN) were sonicated for 5 min using ultrasonic bath and filtered using 0.2 *μ*m membrane filters before used.

#### 2.2.2. Diluting Solution

Diluent *A*: A mixture of 0.1 N HCl and 0.6 M Tris buffer at a ratio of 7 : 3 was prepared and the final pH was adjusted to 8.0 ± 0.5 with 2N HCl or 2N NaOH.

Diluent *B*: Methanol.

#### 2.2.3. Standard Preparation

50.0 mg RPS working standard was accurately weighed and transferred into a clean and dry 100 mL standard volumetric flask and fully dissolved with the diluent *A* and finally volume up to the mark with the same solvent. 

After which, 2 mL aliquot of the above solution was transferred instantaneously into another dry 100 mL standard volumetric flask and diluted to the mark with the diluent *B* to make a concentration of 10 *μ*g/mL. Finally the solution was filtered through 0.45 *μ*m PTFE (polytetrafluoroethylene) disk filter during transfer to the amber vial.

#### 2.2.4. Dissolution Test Conditions and Analytical Procedure

Twelve-vessel dissolution unit was used to analysis of test samples by using this developed method. The dissolution test followed the procedure predetermined by US FDA (United States Food and Drug Administration) using USP apparatus 2 (paddles) [24]. The paddle speed was 100 rpm, and the temperature of the dissolution medium was maintained 37.0 ± 0.5°C by covering the vessel. Dissolution medium was prepared according to the US FDA recommendation. The US FDA recommended dissolution test conditions, at acid stage was; 700 mL of 0.1N HCl for 120 min and at buffer stage, 300 mL of 0.6 M Tris buffer was added into the acid medium with necessary adjusting of pH to 8.0 with 2N HCl or 2N NaOH and the dissolution test was continued for 45 min.

After two hours, 5 mL samples were collected for assay analysis of RPS from acid stage and immediately diluted to 10 mL with the 5 mL of diluent *B* and 5 mL samples were also withdrawn from buffer stage at intervals (10, 20, 30 and 45 min) for assay analysis of RPS and immediately diluted to 10 mL with the 5 mL of diluent *B*. The samples were filtered through 0.45 *μ*m PTFE disk filter and transferred to the amber vial. The first 2 mL of the sample that obtained each time during filtration was discarded in order to clean out the filter.

### 2.3. Method Validation Parameters

#### 2.3.1. System Suitability

To evaluate system suitability of the method, six replicate injections of standard RPS solution were injected, and the percent relative standard deviation (%RSD) values of the parameters such as repeatability, tailing factor, and retention time were calculated in each case.

#### 2.3.2. Linearity

Three replicated, eight different concentration levels test solutions from 0.5 to 120% of analyte concentration (0.05, 0.1, 1.0, 2.5, 5.0, 8.0, 10.0, and 12.0 *μ*g/mL) were prepared from RPS standard. The linearity was evaluated by mean of replicated peak area versus concentration, which was calculated by linear regression analysis.

#### 2.3.3. Specificity

The specificity of the developed RP-HPLC method was investigated by chromatographic analysis of commercially available placebo samples of AcipHex tablets in the usual concentration of excipients. To check the non-interference of placebo, placebo ingredients of the AcipHex tablet formulation but not including RPS were transferred to dissolution vessels containing 1000 mL of diluent *A* and stirred at 37°C for 120 min at 100 rpm using USP paddle apparatus. While aliquots of the solution was filtered through Whatman number 42 filter paper and pipetted 5 mL into a 10 mL volumetric flask and volume up to the mark with diluent *B*, the 3D image of peak and the peak purity tool was used to evaluate the peak purity of the dissolution test solution.

#### 2.3.4. Accuracy

Accuracy parameter was determined by the recovery test, which consisted of adding and dissolving known amounts of RPS into the placebo sample solutions with followed by required dilution in methanol. Placebo solution was prepared by dissolving placebo samples in 1000 mL of diluent *A* at 37°C, and after which, filtered through Whatman number 42 filter paper. This test was conducted by four different concentrations (0, 50, 100, and 120%), that is, 0, 5.0, 10.0, and 12.0 *μ*g/mL of test sample in three replicate sample preparations and the percent recoveries (mean ± %RSD of three replicates) of RPS in drug-placebo form were calculated.

#### 2.3.5. Precision

Precision of the method was determined by repeatability (intraday precision) and intermediate precision (interday precision) of RPS standard solutions. Intra-day precision was determined in six replicates of RPS standard solution (10 *μ*g/mL) on the same day. The intermediate precision of the method was also evaluated on different days with different analyst using the same column and instrument in the same laboratory. The results were expressed as %RSD of the measurements. 

#### 2.3.6. Stability of Solution

The solution stability was tested by allowing the prepared dissolution assay tested sample (sample was withdrawn at buffer stage) with to stand exposed to room light and ambient room temperature for 6 hours. The sample was injected every hour, and different percentage was compared to the value of initially prepared original sample solutions.

#### 2.3.7. Sensitivity

For sensitivity study the limit of detection (LOD) and limit of quantitation (LOQ) were estimated by determination of signal-noise ratios of 3.3 : 1 and 10 : 1, respectively, by injecting series of dilute solution with known concentrations. 

#### 2.3.8. Robustness

To determine the robustness of the current method, the effect of flow rate was studied at 0.9 and 1.1 mL/min instead of 1.0 mL/min. The effect of mobile phase composition was assessed at (buffer : ACN = 59 : 41, v/v) and (buffer : ACN = 61 : 39, v/v) instead of (buffer : ACN = 60 : 40, v/v). The effect of wavelength change was studied at 279 nm and 282 nm instead of 280 nm.

#### 2.3.9. Ruggedness

Ruggedness of the current method was determined by analyzing six dissolution assay sample solutions of AcipHex tablet by different Instrument (Dionex ultimate 3000 series HPLC), different column of the same brand (ProntoSIL SC, C 18-ace-EPS, 100 × 4.6 mm ID and 10 *μ*m particle size) but in the different laboratory to check the reproducibility of the test result.

## 3. Results and Discussion

### 3.1. Method Optimization

To avoid the degradation of RPS the buffer solution pH of mobile phase was adjusted at 7.6, as RPS, is most stable at higher pH. The mobile phase was optimized in the ratio of buffer : ACN (60 : 40 v/v) with a flow rate 1.0 mL/min. Chromatographic separation was achieved at 280 nm, where the linearity of the proposed method was found more satisfactory rather than RPS maximum UV absorbance (*λ* max) at 285 nm. Regular commercial C 18 column of ProntoSIL SC, C 18-ace-EPS, 100 × 4.6 mm ID containing particle size of 3, 5, and 10 *μ*m was tried. The peak was sharp and tailing factor was acceptable for the column of 3, 5 and 10 *μ*m size particles but 10 *μ*m particle size column life was found interestingly more stable at this high pH. The inject volume was chosen only 20 *μ*L, and the total run time was fixed at 4 min as further any peak of excipients, or blank was not observed.

While developing the method, stability of RPS into US FDA recommended Tris buffer (pH 8.0) solution was taken into account sincerely. RPS was found less stable in tris buffer medium. To enhance stability of RPS, an instant dilution was performed into the organic solvent (methanol), all through the preparation of standard and sample. US FDA suggests additional 0.5 N NaOH into the dissolution medium to stabilize the sample. Additional NaOH may increase the pH of tris buffer medium, this part was avoided in this study to maintain the pH of dissolution medium at 8.0, and HPLC column life is also very shorter at high pH. Moreover, according to USP and US FDA, pH of the dissolution medium as buffered aqueous solution usually is 4 to 8, and a higher pH should be justified, and in general, should not exceed pH 8.0 [23, 25]. 

### 3.2. Method Validation

#### 3.2.1. System Suitability

The results (mean ± %RSD of six replicates) of the chromatographic parameters of the proposed method indicate the good performance of the system (Table 1).

#### 3.2.2. Linearity

The linearity of the calibration plot for the method was obtained over the calibration ranges tested, that is, 0.05–12 *μ*g/mL (Table 2), and the correlation coefficient obtained was >0.999, thus indicating high degree of correlation between peak areas and concentrations of the analyte. The regression equation for RPS was *y* = 20235*x* − 432 (*R* ^2^ = 0.9999). 

#### 3.2.3. Specificity

**Figure 2:**
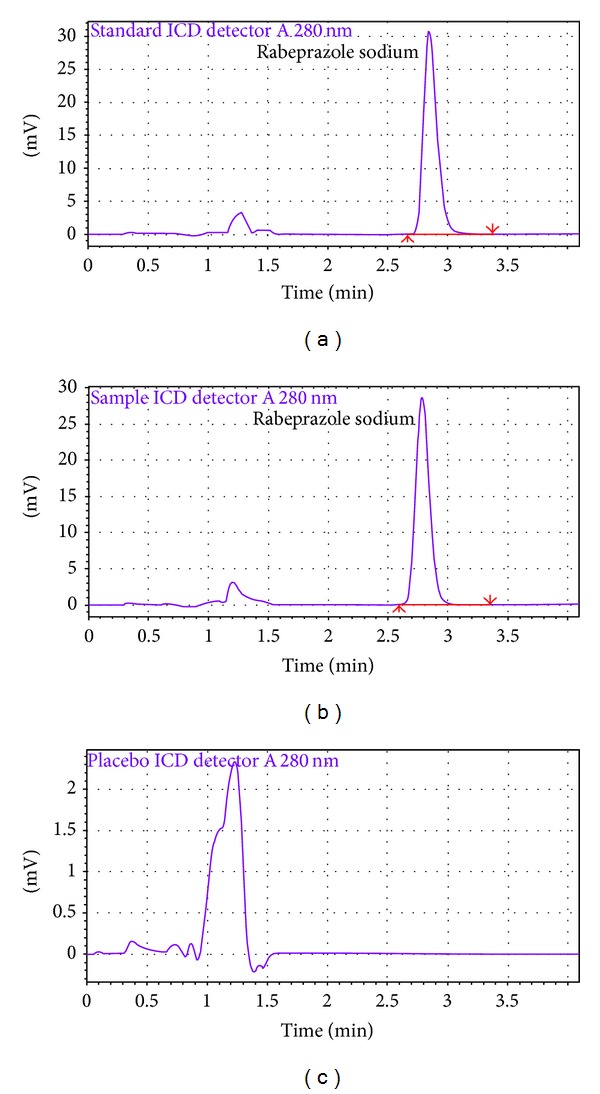
RP-HPLC chromatogram of (a) standard, (b) assay sample, and (c) placebo sample of rabeprazole sodium.

**Figure 3:**
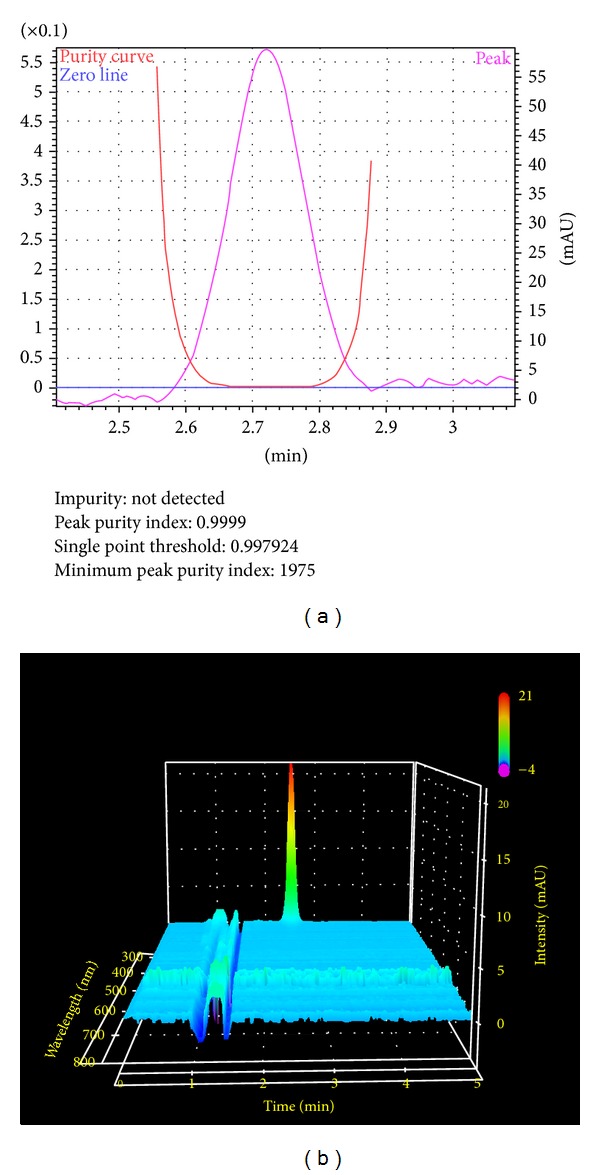
Peak purity of dissolution assay sample of rabeprazole sodium. (a) peak purity curve (b) In 3D visualization: peak retention time (min), absorbance (nm), and intensity (mAU) are presented in *X*-, *Y*-, and *Z*-axes, respectively, from Shimadzu LabSolution software.

The chromatograms of blank, placebo, test sample, and standard, used to justify the specificity of target analyte. The method was specific since none of peaks did not appear at the retention time of RPS (Figure 2) and in every case the peak purity was 99.99% and no impurity peaks were detected (Figure 3). 

#### 3.2.4. Accuracy

The overall results of percent recoveries (mean ± %RSD) of drug-placebo solutions ranged from 99.9 to 101.9 are indicating good accuracy of the proposed RP-HPLC method (Table 3). The results also revealed that there was no interference of excipients. 

#### 3.2.5. Precision

The values of %RSD for intra-day and inter-day variation were found very well and within 2% limit, indicating that the current method is precise (Table 4).

#### 3.2.6. Stability of Solution

In the stability study, the peak area of RPS in dissolution assay sample solutions decreased insignificantly at four hour which is more than 2.0% from initial. This indicates that sample solutions were stable for at least 3 hours, which was absolutely sufficient to complete the analytical procedure (Table 5). 

#### 3.2.7. Sensitivity

The LOD and LOQ by the proposed method were found for RPS that was 0.01 *μ*g/mL and 0.03 *μ*g/mL, respectively.

#### 3.2.8. Robustness

The effects of robustness study under different altered conditions of this proposed method were satisfactory. The mean and %RSD of standard and analyzed sample indicate that the current method is robust (Table 6).

#### 3.2.9. Ruggedness

**Figure 4:**
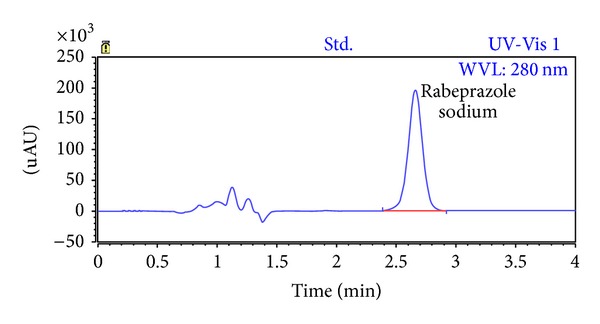
Chromatogram of rabeprazole sodium from Dionex HPLC 3000 Series.

The results (% of recovery ± RSD) of six assay samples are indicating the ruggedness of the current method (Figure 4, Table 7).

### 3.3. Application of the Developed Method

The validated method was used for the analysis of a commercial brand (AcipHex) of RPS delayed-release tablet formulation with dose strength 20 mg per tablet. To achieve significant results total twenty-four numbers of tablets were analyzed in two different days (12 tablets per day) into the twelve vessels containing dissolution unit, and the sampling was done from the acid and the buffer stage according to the US FDA recommended time intervals. At acid stage, there was always 0% release of RPS. While at buffer stage, the plateau of the dissolution profile was reached within 20 min for both days analysis. Mean release at buffer stage of twelve tablets was found 99% with %RSD 1.7 and 100% with %RSD 1.1 for day 1 and day 2, respectively at 20 min. 

**Figure 5:**
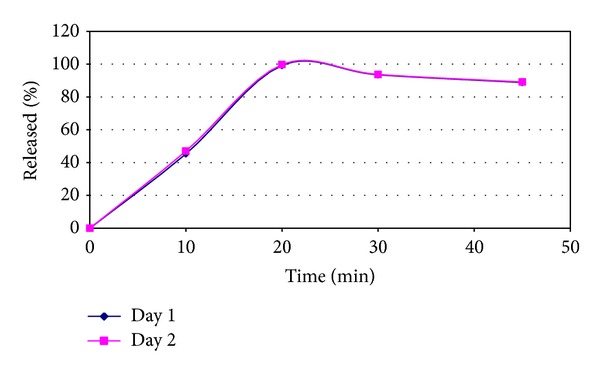
Dissolution profile of AcipHex DR tablets in tris buffer medium.

Two-days analysis results of AcipHex DR tablets also agreed with the stability nature of RPS in tris buffer. The RPS release for AcipHex was found rapid and maximum (98–102%) at 20 min, most of all the tablets disintegrated within 10 min. After reaching maximum release; significant degradation was observed at 30 min and 45 min in tris buffer medium containing sensitive RPS sample (Figure 5). The mean release of RPS (*n* = 12) on day 1, at 30 min and 45 min was found 94% (%RSD 2.1) and 89% (%RSD 1.6) and on day 2, at 30 min and 45 min the mean release of RPS was found 93% (%RSD 0.9) and 87% (%RSD 1.1) respectively. 

The US FDA guideline for dissolution testing of extended release oral dosage forms that recommends the last point should be the time point where 80% drug has dissolved [26]. Finally, during dissolution assay preparations, sample was withdrawn from buffer medium at 20 min, while it released maximum, and after immediate dilution into methanol the assay sample was found stable up to 3 hours.

## 4. Conclusion

In recent days, more importance has been placed on dissolution testing by the pharmaceutical industry and regulatory authorities. The main objective of the proposed method was to establish a reliable procedure to analysis of RPS dissolution samples according to US FDA recommended dissolution medium. The new RP-HPLC method for quantification of RPS is simple, precise, accurate, reproducible and rapid. The developed method was validated based on ICH and USP guidelines and successfully applied to *in vitro* dissolution test studies of RPS. The short run time of 4 min of this method allows to analyze sensitive RPS samples in a short period of time, which also makes it cost-effective for the routine quality control analysis work.

## Supplementary Material

According to ICH (International Conference on Harmonization) Q2 (R1) and the USP (United States Pharmacopeia), the linearity of an analytical procedure is its ability (within a given range) to obtain test results which are directly proportional to the concentration (amount) of analyte in the sample and normally calculated by using an appropriate least-squares regression program. Typically, a square of the correlation coefficient (R2 ≥ 0.98) demonstrates linearity. In this proposed RP-HPLC method the linear regression equation for RPS was found, y = 20,235x – 432, (R2 = 0.9999) by plotting peak area (y) versus the concentration (x). The results show that an excellent correlation exists between peak areas and concentrations of the drug.

## Figures and Tables

**Table 1:** Chromatographic characteristics of system suitability study.

Parameters	Value (mean ± %RSD), *n* = 6
Peak area	206343 ± 0.2
Tailing factors	1.1 ± 1.5
Retention time (min)	2.7 ± 0.1

**Table 2:** Linearity of the method.

Number of preparation	Sample conc. %	Sample conc. (*μ*g/mL)	Peak area	Mean of peak area
1	0.50	0.05	1080	1047
			1028	
			1032	
2	1.00	0.10	2101	2053
			1987	
			2072	
3	10.00	1.00	18948	18421
			18171	
			18145	
4	25.00	2.50	49348	50105
			50589	
			50378	
5	50.0	5.00	101227	101425
			101704	
			101345	
6	80.0	8.00	160938	161103
			161536	
			160835	
7	100.0	10.00	202514	202551
			202813	
			202325	
8	120.0	12.00	244146	241939
			240250	
			241420	

**Table 3:** Accuracy studies of RPS in drug-placebo solutions.

Sample conc. %	Amount added (*μ*g/mL)	Peak area of sample	Amount recovered (*μ*g/mL)	% Recovery	% Recovery (mean ± %RSD)
0.0	0.0	0.0	0.0	0.0	0.0
	0.0	0.0	0.0	0.0	
	0.0	0.0	0.0	0.0	
50	5.0	97466	5.1	101.9	101.7 ± 0.4
	5.0	96848	5.0	101.2	
	5.0	97472	5.1	101.9	
100	10.0	193783	10.1	101.3	101.4 ± 0.2
	10.0	194406	10.1	101.6	
	10.0	193829	10.1	101.3	
120	12.0	229764	12.0	100.1	100.0 ± 0.1
	12.0	229820	12.0	100.1	
	12.0	229235	12.0	99.9	

**Table 4:** Intraday and interday precision of the method.

Sr. number	Concentration (*μ*g/mL)	Peak area of standard solution	
		Intraday precision	Interday precision
1	10	206799	210365
2	10	205732	209625
3	10	203999	211009
4	10	202773	210253
5	10	208159	209663
6	10	210564	206186

Mean		206338	209517
%RSD		1.4	0.8

**Table 5:** Stability of analytical sample solution.

Hour	Concentration (*μ*g/mL)	Room temperature (°C)	Peak area of sample	Difference percentage (%) from initial
0	10	25 ± 2	207960	0.0
1	10	25 ± 2	205734	1.1
2	10	25 ± 2	204729	1.6
3	10	25 ± 2	204185	1.8
4	10	25 ± 2	202898	2.4
5	10	25 ± 2	200037	3.8
6	10	25 ± 2	197381	5.1

**Table 6:** Robustness of the method.

Parameters	Variance	Amount added (*μ*g/mL)	Retention time (min)	Mean area of peak ± %RSD, *n* = 3	
				Standard	Sample
Flow rate	0.9 mL/min	10	2.8	219467 ± 0.3	225203 ± 0.4
	1.1 mL/min	10	2.2	184626 ± 0.3	188516 ± 0.3
Organic (%) in mobile phase	59	10	2.7	207066 ± 0.5	206049 ± 0.3
	61	10	2.5	203858 ± 0.3	203051 ± 0.4
Detector wavelength	278 nm	10	2.7	199421 ± 0.2	198228 ± 0.6
	282 nm	10	2.7	213519 ± 0.4	211768 ± 0.7

**Table 7:** Ruggedness of the method.

Sample	Amount of standard RPS (mg)	Drug release (%) in buffer at 20 min (mean ± %RSD), *n* = 6
AcipHex 20 mg DR tablet	20.0	100.1 ± 0.1
