# Supplementary material for: Validation and Application of a New Reversed Phase HPLC Method for In Vitro Dissolution Studies of Rabeprazole Sodium in Delayed-Release Tablets
Source: J Anal Methods Chem. 2013 Aug 22;2013:976034. doi: 10.1155/2013/976034 (PMC3766580; doi:10.1155/2013/976034)
Supplement: Supplementary file 1 — According to ICH (International Conference on Harmonization) Q2 (R1) and the USP (United States Pharmacopeia), the linearity of an analytical procedure is its ability (within a given range) to obtain test results which are directly proportional to the concentration (amount) of analyte in the sample and normally calculated by using an appropriate least-squares regression program. Typically, a square of the correlation coefficient (R2 ≥ 0.98) demonstrates linearity. In this proposed RP-HPLC method the linear regression equation for RPS was found, y = 20,235x – 432, (R2 = 0.9999) by plotting peak area (y) versus the concentration (x). The results show that an excellent correlation exists between peak areas and concentrations of the drug. [file 976034.f1.pdf]

SUPPLEMENTARY MATERIAL

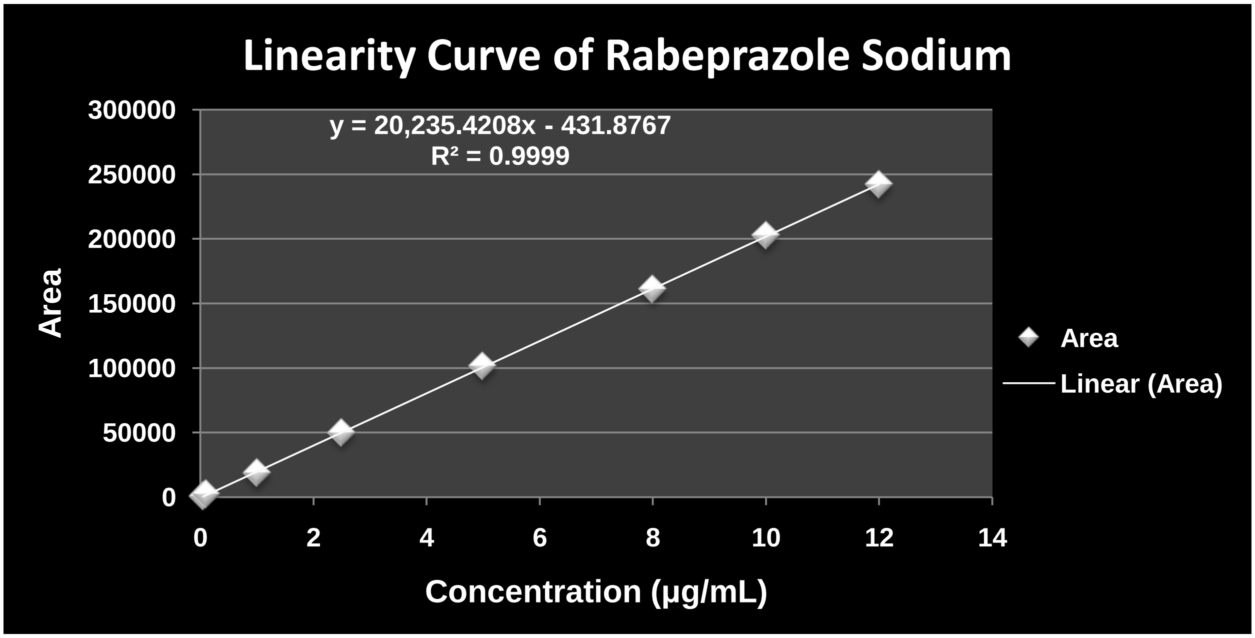

In this proposed RP-HPLC method the linear regression equation for RPS was found, y = 20,235x – 432, (R^2 = 0.9999) by plotting peak area (y) versus the concentration (x). The results show that an excellent correlation exists between peak areas and concentration of drugs.
